# WO_3_/Buckypaper Membranes for Advanced Oxidation Processes

**DOI:** 10.3390/membranes10070157

**Published:** 2020-07-20

**Authors:** Giovanni De Filpo, Elvira Pantuso, Aleksander I. Mashin, Mariafrancesca Baratta, Fiore Pasquale Nicoletta

**Affiliations:** 1 Department of Chemistry and Chemical Technologies, University of Calabria, 87036 Rende (CS), Italy; mariafrancesca.baratta@unical.it; 2 Department of Pharmacy, Health and Nutritional Sciences, University of Calabria, 87036 Rende (CS), Italy; elvirapnt.ep@gmail.com; 3 Applied Physics & Microelectronics, Lobachevsky State University of Nizhni Novgorod, 603950 Nizhni Novgorod, Russia; mashin@unn.ru

**Keywords:** chemical vapor deposition, buckypapers, single wall carbon nanotubes, tungsten trioxide, photocatalysis, membranes

## Abstract

Photocatalytic materials, such as WO_3_, TiO_2_, and ZnO nanoparticles, are commonly linked onto porous polymer membranes for wastewater treatment, fouling mitigation and permeation enhancement. Buckypapers (BPs) are entanglements of carbon nanotubes, which have been recently proposed as innovative filtration systems thanks to their mechanical, electronic, and thermal properties. In this work, flexible membranes of single wall carbon nanotubes are prepared and characterized as efficient substrates to deposit by chemical vapor deposition thin layers of WO_3_ and obtain, in such a way, WO_3_/BP composite membranes for application in advanced oxidation processes. The photocatalytic efficiency of WO_3_/BP composite membranes is tested against model pollutants in a small continuous flow reactor and compared with the performance of an equivalent homogeneous WO_3_-based reactor.

## 1. Introduction

Advanced oxidation processes, AOPs, allow the mineralization of organic pollutants by the generation of highly reactive hydroxyl radicals [1]. Photocatalytic reactions are particular AOPs, which are carried out when a catalyst is irradiated by a radiation of suitable wavelength [2]. 

Photocatalysis finds several interesting applications including selective organic reactions, pollutant degradation, photocatalytic surfaces (e.g., tiles, cements, paints, and asphalts), filters for air purification, water splitting in H_2_ and O_2_, water purification plants, CO_2_ reduction to energy fuels and bacterial disinfection [3,4,5,6,7,8]. Semiconductors are the most common used materials used in photocatalytic processes. Upon irradiation, electrons are promoted from the valence band to the conduction band, generating electron–hole pairs. Electrons and holes can move to the semiconductor surface and generate oxidizing species such as hydroxyl radicals (OH^●^), superoxide anions (O_2_^−●^) and hydrogen peroxide molecules (H_2_O_2_), which are able to react with the present chemical species (dyes, pollutants, and other undesired molecules) and degrade them [9]. Several materials have photocatalytic properties (GaP, GaAs, CdSe, CdS, Fe_2_O_3_, TiO_2_, WO_3_, ZnO, SnO_2_, and CdS, just to mention a few), however not all of them are sufficiently efficient and stable over time to be used. In fact, GaP, GaAs, CdSe, CdS, or Fe_2_O_3_ are less stable in the air and degrade more easily. ZnO forms a passivating layer of Zn(OH)_2_ on its surface, which seriously compromises its photocatalytic properties [9]. Another important factor determining the choice of a semiconductor is its band-gap value, which must be as small as possible in order to allow the use of electromagnetic radiation with larger wavelengths. Titanium dioxide, TiO_2_, and tungsten trioxide, WO_3_, are low-cost semiconductor materials characterized by reduced toxicity towards environment and health and relatively low energy band-gaps (3.2 eV and 2.6 eV, respectively), which allow their activation with UV-Vis light (387 nm and 476 nm, respectively) [10].

Further problems for the use of semiconductors in the photocatalytic processes are:

1. The need of high surface area, which can be overcome by using nanometer sized materials; and
2. The semiconductor recovery after their use, which can be solved, for example, by nano-semiconductors with a magnetic core, by chemically cross-linked semiconductor nanoparticles onto polymer or ceramic membranes and, more recently, by vapor deposition of thin semiconductor films onto suitable substrates [11].

Thin film deposition methods can be distinguished in physical vapor deposition, PVD, and chemical vapor deposition, CVD, processes. In both methods, atoms or molecules in their vapor phase are carried onto the substrate surface and settle to form a thin layer [11].

Food, pharmaceutical, and, more in general, chemical plants need efficient separation and purification processes in order to guarantee an efficient treatment of their wastewaters. The removal of toxic pollutants from industrial wastewaters is a challenge due to the facts that they could not be effectively removed by filtration, adsorption, sedimentation, bio-oxidation, chlorination, coagulation, UV, and other classical treatments [12,13] and could represent potential threats to environment and health even at low concentrations (few ng L^−1^) as a consequence of their bio-accumulation [14,15].

Recently, polymer membranes have been suggested as simple and efficient materials to be used in water treatments including separation, purification, desalinization, recovery of critical raw materials and AOPs [16,17,18,19]. In fact, photocatalytic membranes, i.e., membranes with embedded or supported semiconductors by chemical binding, physical blending and vapor deposition, have been prepared in order to reduce/mitigate membrane fouling [20,21], enhance filtration fluxes [22], degrade wastewater pollutants, and remediate the concentrate [23]. Nevertheless, the binding of photocatalysts and polymer functionalization could need several chemical reactions and long cleaning processes, while the physical blending generally alter the mechanical properties of membranes and reduce the photocatalytic performance, as only the catalysts onto the membrane surface can play their photocatalytic activity.

In addition to their potential applications as TV screens, fire protection systems, heat dispersion in microelectronics, electrical-conductive tissue engineering, electromagnetic interferences shielding, electrodes for batteries and supercapacitors, buckypapers (BPs) have been proposed as innovative, high-temperature resistant and lightweight filtration systems. They consist of an entangled assembly of carbon nanotubes (CNTs) obtained by filtration of CNT dispersions through a polymer membrane [24,25]. According to such simple procedure, it is possible to get large-scale BP membranes that merge the mechanical, electronic, and thermal properties of CNTs with the flexibility, porosity, and transport properties of polymer membranes [26]. At a microscopic level, BPs show a highly disordered porous morphology due to π–π and van der Waals interactions between and within bundles of carbon nanotubes [27,28]. Consequently, BPs can result brittle. Such a problem and the risk of nanotubes release can be overcome enhancing the mechanical properties of BPs by polymer intercalation [29,30].

In this work, flexible membranes of single wall carbon nanotubes, SWNT, (or buckypaper, BP) were prepared and characterized as efficient substrates to deposit by CVD thin layers of WO_3_ and obtain, in such a way, WO_3_/BP composite membranes for application in advanced oxidation processes. The photocatalytic efficiency of WO_3_/BP composite membranes was tested against model pollutants (Methylene Blue, Indigo Carmine, and Diclofenac Sodium) in a small continuous flow reactor and compared with the performance of an equivalent homogeneous WO_3_-based reactor.

## 2. Materials and Methods

### 2.1. Preparation of BP Membranes

Buckypaper membranes were obtained by filtration of SWNT dispersions through PTFE disks (diameter = 47 mm, average pore size = 5 μm, Durapore^©^, Merck KGaA, Darmstadt, Germany). The average diameter of SWNTs was 1.4 ± 0.1 nm and their length was longer than 5 µm as reported in the datasheet from Sigma-Aldrich, Milan, Italy. Thirty milligrams of SWNTs were dispersed in 200 mL of a 0.4% TRITON X100 water solution by an ultrasonic bath (model M1800H-E, Bransonic, Danbury, CT, USA) for 30 min. Then, solutions were filtered through the PTFE disks with a vacuum pump (pressure = −0.04 bar), washed with 20 mL of methanol and, finally, dried at room temperature. All chemicals were purchased from Sigma-Aldrich, Milan, Italy.

### 2.2. Deposition of WO_3_ onto BP Membranes

The deposition of nanostructured tungsten trioxide onto BP membranes was obtained by reactive RF sputtering using of a tungsten target (purity 99.999%, Goodfellow Cambridge Ltd., Huntingdon, England) in the presence of oxygen (purity 99.999%) as process and reactive gas under different conditions of oxygen flow, sample-target distance, sputtering time, and applied RF process power. The optimal process conditions in term of layer homogeneity and catalyst droplet size were found to be: Oxygen flow 35 mL min^−1^, sample-target distance 8 cm, sputtering time 30 min, applied RF process power 50 W.

The amorphous WO_3_ thin films deposited on BP membranes were converted in monoclinic WO_3_ thin films, which are characterized by a larger catalytic activity, by heat treatment at 350 °C for 30 min.

### 2.3. Characterization of BP and WO_3_/BP Membranes

The porosity, P, of BP and WO_3_/BP membranes was determined by gravimetric method at 25 °C, measuring the weight of a wetting liquid (3M-FC-40, 3M Italia Srl, Pioltello, Milan, Italy), contained in the membrane pores. The porosity was calculated according to the following Equation (1):

(1) $$P=\frac{\frac{w_{w}-w_{d}}{d_{w}}}{\frac{w_{w}-w_{d}}{d_{w}}+\frac{w_{d}}{d_{m}}}$$

where w_w_ is the weight of the wet samples, w_d_ the weight of dry samples, d_w_ the wetting liquid density (1.855 g·cm^−3^), and d_m_ is the average membrane density (0.60 ± 0.03 g·cm^−3^ as calculated from measurements of buckypaper weight, thickness, and surface area).

Pore size distribution was evaluated by a capillary flow porometer (CFP-1500 AEXL, PMI Porous Materials Inc., Ithaca, NY, USA). Membranes were fully wetted by keeping them in Porewick^®^ (Sigma-Aldrich, Milan, Italy) for 24 h. Then, nitrogen was gradually allowed to flow into the membrane by increasing its pressure and the registration of gas pressure and permeation flow rate allowed the calculation of the pore size distribution. 

The electrical conductivity of membranes was determined from the BP electrical resistance in I–V (current–voltage) measurements by a waveform generator (33220A Agilent, Santa Clara, CA, USA) and a digital multimeter (34410A Agilent, Santa Clara, CA, USA) on BP rectangular strips (width 5 mm and length 3 cm).

The mechanical properties were measured with a Sauter TVO-S tensile tester equipped with a Sauter FH-1k digital dynamometer and AFH FAST software (Sauter GmbH, Balingen, Germany). The rectangular strips (width 5 mm and length 3 cm) were tested at a strain rate of 0.1 mm·min^−1^. The tests allowed the determination of the tensile strength as the maximum stress, the fracture strain as the percentage of elongation at the breaking point, and the Young’s modulus.

Thermogravimetric analysis (TGA, STA 2500 Regulus simultaneous thermal analyzer, Netzsch, Selb, Germany) was employed to assess the BP membrane thermal stability. TGA was carried out from room temperature to 750 °C with a heating rate of 5 °C/min in a flowing gas mixture consisting of 1% O_2_ and 99% Ar at a flow rate of 100 sccm. The average roughness of WO_3_/BP surfaces was evaluated by atomic force microscopy (Nanoscope III, Digital Instruments, Santa Barbara, CA, USA). Static contact angle measurements of BP and WO_3_/BP membranes were measured with a goniometer (Nordtest, Serravalle Scrivia AL, Italy) at 25 °C. A drop (2 µL) of water was put onto the sample surface by a micro-syringe and measurements were carried out by setting the tangents on both visible edges of the droplet on five different positions of each sample and calculating the average value of the measurements.

The permeation tests were carried out with distilled water using a filtration cell having an active area of 5 cm^2^. The feed solution at 25 ± 1 °C was pumped by a gear pump at a transmembrane pressure of 1 bar. The feed flow rate was 250 mL·min^−1^. Permeate samples were collected every 5 min in order to determine the transmembrane flux, J, defined as:

(2) $$J=\frac{V_{p}}{A\Delta t}$$

where V_p_ was the permeate volume passed through the membrane in the fixed time interval, Δt, and A was the effective membrane area.

### 2.4. Photodegradation Experiments

The photoactivity of WO_3_/BP membranes was investigated in a small continuous plant with model pollutant water solutions (250 mL) of a cationic dye (Methylene Blue, MB, 5, 10, and 20 ppm), an anionic dye (Indigo Carmine, IC, 20 ppm) and a drug (Diclofenac Sodium, DS, 20 ppm), which were recirculated by a peristaltic system (flow rate 16.6 mL·min^−1^, Masterflex^®^ L/S^®^, Cole-Parmer Srl, Cernusco sul Naviglio, MI, Italy) through a round cell. All model pollutants were purchased from Sigma-Aldrich, Milan, Italy. The experiment temperature was 25 ± 1 °C being the becker with the pollutant solutions placed in a thermostatic bath (model 1225, VWR, Milan, Italy), which kept constant the flowing solution temperature and avoided the pollutant thermolysis. All model pollutans were purchased from Sigma Aldrich, Milan, Italy. The WO_3_/BP membranes divided the cell volume in two compartments: The upper one (thickness 5 mm, photocatalytic area 8 cm^2^, irradiated volume 4 cm^3^) was equipped with a N-BK7 optical glass window to allow UV-Vis irradiation from a Suntest CPS+ sun simulator (1.5 kW Xenon arc lamp, with an average irradiance of 500 W·m^−2^ in the wavelength range 300 nm–800 nm, see Figure S1 of SI, Atlas, Linsengericht-Altenhaßlau, Germany). The light power of sun simulator was calibrated by a FieldMaxII-TO digital power/energy meter (Coherent Italia S.r.l., Monza, Italy) equipped with a PM10 thermopile. The WO_3_ sputtered surface of membranes was exposed to UV light. After irradiation, the solution passed through a quartz flow cuvette placed inside a spectrophotometer able to read at regular intervals (5 min) the absorbance value at the maximum absorption wavelength of MB (665 nm), IC (610 nm), and DS (275 nm). Similarly, the photoactivity of 0.2 mg of monoclinic WO_3_ nano-powder (which was the same amount of WO_3_ sputtered onto BP membranes) was measured. As each experiment generally lasted 150 min and the recirculation time was around 15 min, the average contact time of solutions with the active photocatalysis region was extimated in 10 min.

The photodegradation of pollutants [31] is generally described by the first-order kinetics

(3) $$\frac{dC}{dt}=-kC$$

where C is the pollutant concentration, k is the rate constant and t is the reaction time. After integration, the following equation is obtained:

(4) $$ln\frac{C(t)}{C_{0}}=-kt$$

where C_0_ and C(t) are the initial concentration and the concentration at time t of the pollutants. The rate constant can be obtained from the slope of the plot of $ln\frac{C(t)}{C_{0}}$ as a function of t.

Experimental data were corrected by taking into account the effective photon fluence impinging on WO_3_ layer (see SI).

The percentage of pollutant removal, %R, was calculated as:

(5) $$\%R=\frac{C_{0}-C(t)}{C_{0}}\times 100$$

## 3. Results and Discussion

**Figure 1:**
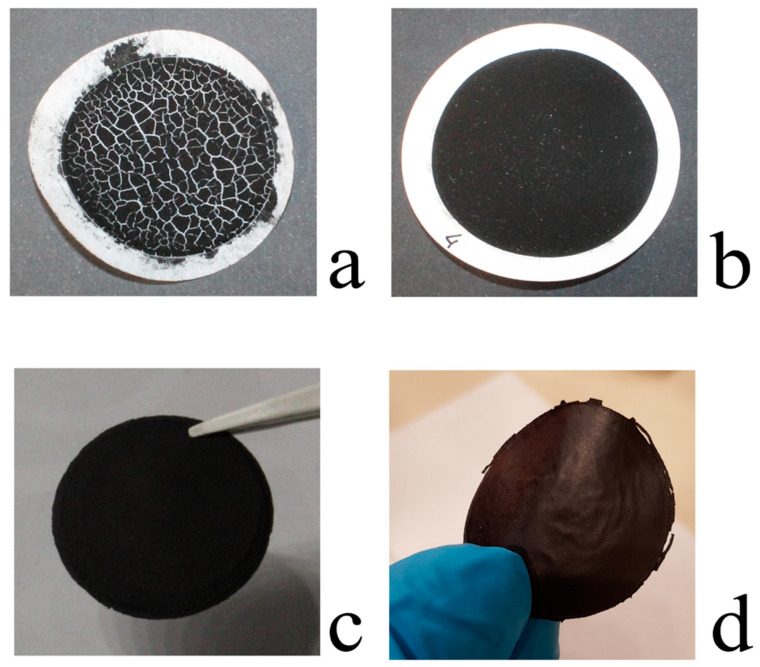
Appearance of buckypaper (BP) membranes under different experimental preparation procedures: (**a**) Brittle and un-detachable BP membrane filtered through a poly (vinylidene fluoride) (PVDF) membrane with reduced pore size; (**b**) whole and detachable; (**c**) free-standing and (**d**) flexible BP membrane obtained under the optimal conditions reported in the Experimental section.

Several factors influence the BP membrane properties including sonication time of the SWNT solution, the magnitude of the vacuum depression used to filter the SWNT solution, the porosity and material of the filtration membranes. After several trials, which gave unacceptable results, including un-detachable BP from polymer membranes (due to small pore size filtration membrane), and brittle and broken BP (due to fast solvent evaporation), Figure 1a, intact BP were obtained under the optimal conditions reported in the Materials and Methods, Figure 1b. Such BPs are easily detachable from the filtration membranes, Figure 1c, and look like free-standing and flexible disks (average thickness 45 ± 2 μm) as shown in Figure 1d. 

**Figure 2:**
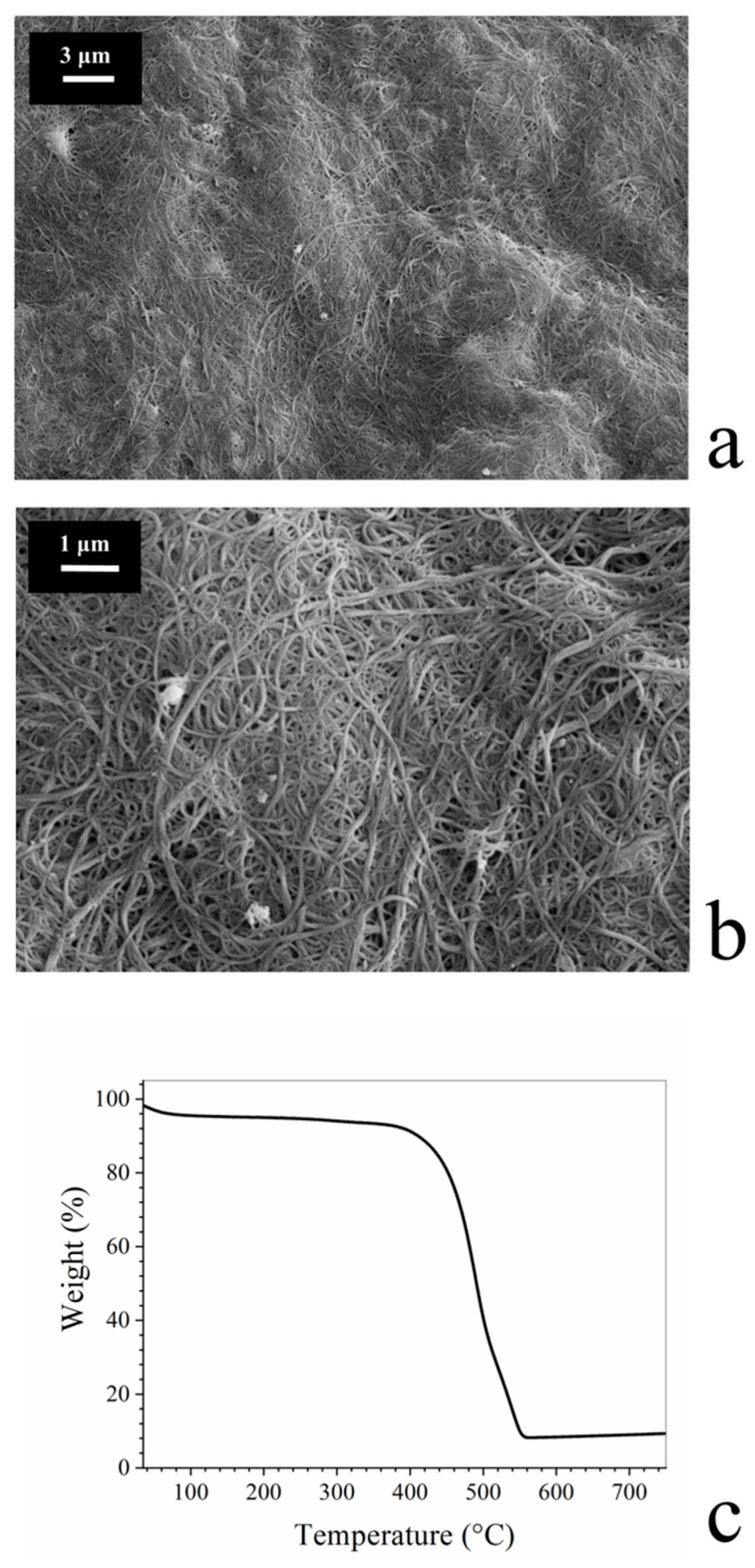
(**a**,**b**) Morphology of BP membranes at two different magnification; (**c**) Thermal stability of BP membranes. The initial weight loss is due to solvent evaporation, while the second one, which starts at around 400 °C, is due to the thermal degradation of single wall carbon nanotubes (SWNTs).

At a microscopic level, BP membranes showed a highly disordered porous morphology due to π–π and van der Waals interactions between and within bundles and clusters of carbon nanotubes, Figure 2a,b. 

The thermal stability of BP membranes was assessed by TGA. As reported in Figure 2c, after an initial weight loss of about 5% due to water evaporation, BP membranes were found to be stable up to 400 °C. For larger temperatures, the degradation of SWNTs is observed.

Table 1 reports some geometrical data and properties for BP membranes. In particular, density, porosity, and water flow rate values of BPs (0.60 ± 0.03 g·cm^−3^, 70 ± 5%, and 12,500 ± 100 L·m^−2^·h^−1^·bar^−1^ respectively) fall in the range of values shown by porous polymer membranes, generally used for filtration processes [32]. In addition, the electrical conductivity and the mechanical properties, namely tensile strenght, fracture strain and Young’s modulus, reported in Table 1, allow to consider BPs as strong and conductive membranes [33].

In order to give photocatalytic properties to BP membranes, they were covered with thin layers of WO_3_ by RF magnetron chemical vapor deposition. CVD is a well-known chemical process for the deposition of desired thin films onto substrate surfaces by chemical reactions among one or more volatile precursors. Nevertheless, the film quality is strongly dependent on the CVD process parameters. Consequently, in this work different sputtering conditions (oxygen flow, sample-target distance, sputtering time, and power) were tested in order to find the optimal set of parameters able to give a homogeneous BP coverage without cracks and small WO_3_ nanoparticles to avoid BP membrane occlusion and increase photoactivity. 

**Figure 3:**
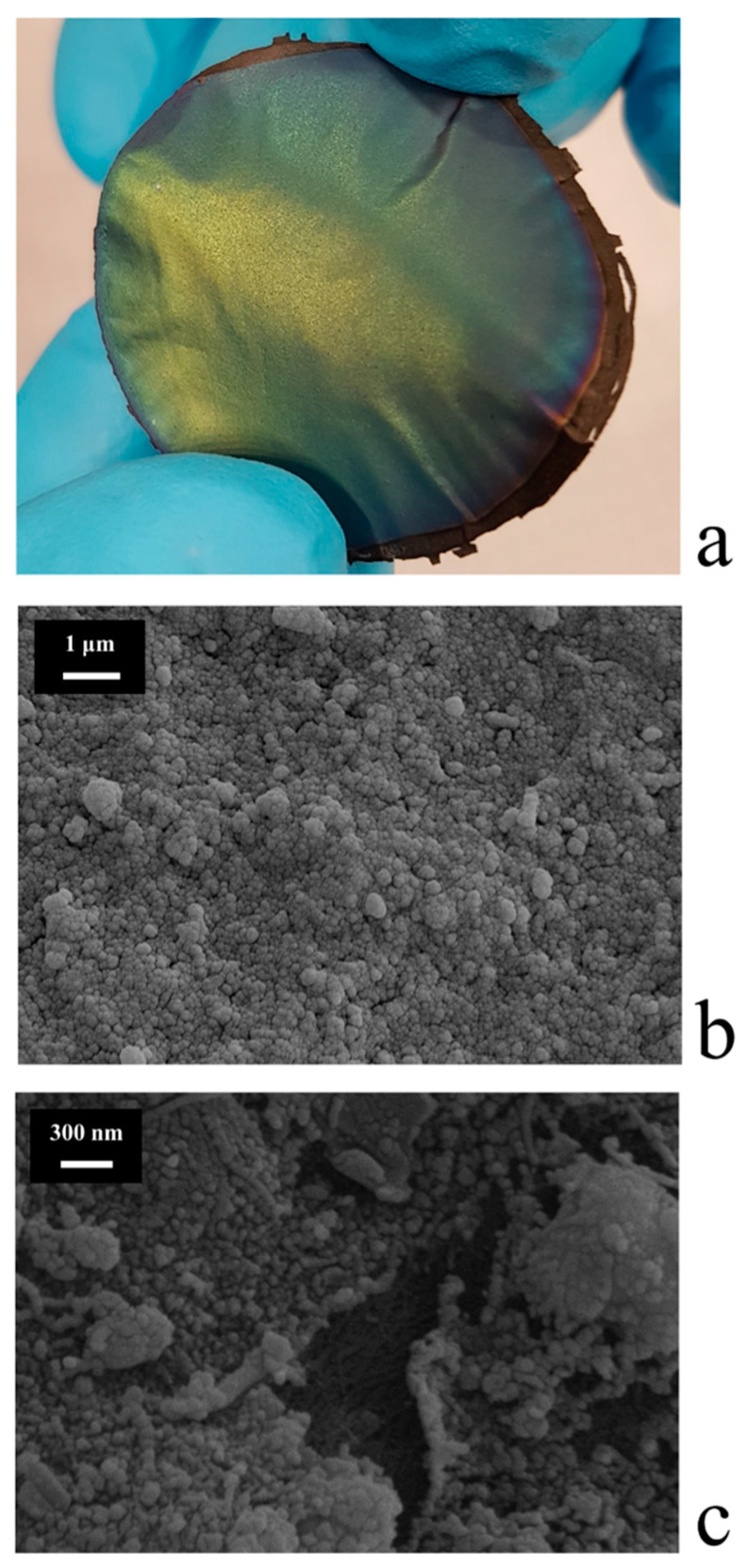
(**a**) Flexible, greenish/yellowish WO_3_/BP membranes; (**b**) Morphology of the top surface of a WO_3_/BP. The WO_3_ coating is a homogeneous layer of small nanoparticles, without cracks and pore occlusion; (**c**) Crack of a faulty WO_3_/BP membrane, inside which it is possible to see the texture of BP substrate.

The best results, in terms of both coverage quality and nanoparticle size, were obtained with the following conditions: Flow(O_2_) = 35 mL·min^−1^, d = 8 cm, t = 30 min, power = 50 W. The WO_3_/BP membranes looked like flexible, greenish/yellowish disks as shown in Figure 3a.

Figure 3b shows the morphology of the top surface of a WO_3_/BP membrane sputtered under the experimental conditions previously reported. A homogeneous layer of small nanoparticles, with a rather spherical shape and an average diameter of around 50 nm, constitutes the WO_3_ coating without cracks and pore occlusion. It is important to remind the presence of BP membrane under the WO_3_ layer. Figure 3c shows the picture of a particular faulty WO_3_/BP membrane with a small crack, where it is possible to see inside the crack the texture of SWNT bundles, which form the BP substrate.

**Figure 4:**
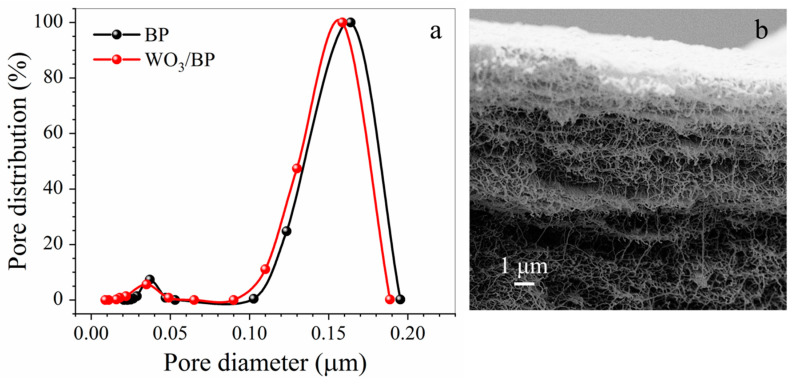
(**a**) Pore size distribution of both a BP membrane (black dots) and a WO_3_/BP photocatalytic membrane (red dots); (**b**) SEM cross section picture of a WO_3_/BP membrane.

Figure 4a,b report the pore size distribution of both a pristine BP and a WO_3_/BP photocatalytic membrane and the SEM cross section picture of a WO_3_/BP membrane, respectively. As shown in Figure 4a both membranes show similar pore size distribution (within experimental errors) with two size populations placed at around 0.160 μm (0.163 ± 0.016 μm and 0.155 ± 0.018 μm, respectively) and at around 0.035 μm (0.037 ± 0.003 μm and 0.035 ± 0.003 μm, respectively) accounting for the presence of a major macroporous structure and a mesoporous structure (inter-tube pores with a size between 2 and 50 nm) formed between SWNT criss-crossings in the sample [34].

It is evident from the cross section of WO_3_/BP membrane, that the WO_3_ layer is a few tens of nanometers thick, but WO_3_ nanoparticles penetrate the BP membrane for ≈ 2 μm, accounting, most likely, for the reduction in the mesopore average size.

However, all the membranes prepared are characterized by a porosity of 70 ± 5%, which is expected to favour the water permeability through them.

**Figure 5:**
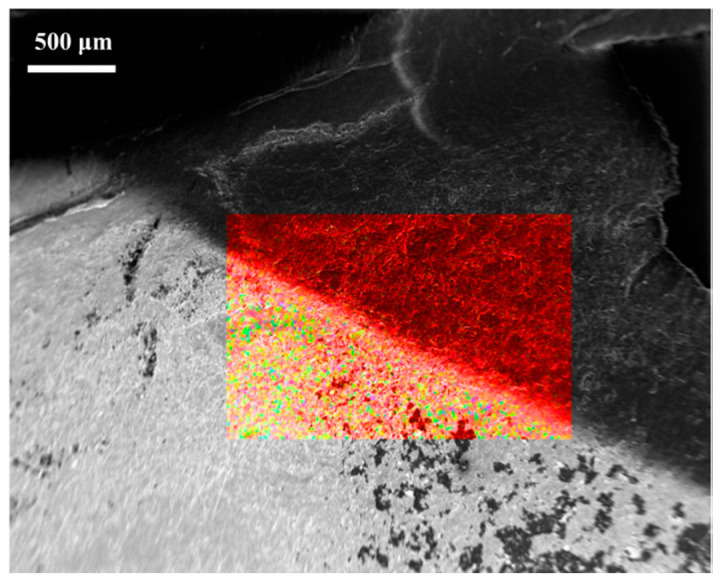
EDX color mapping image and EDX spectra of the border area between a WO_3_/BP membrane (lower area) and a BP membrane (upper area).

The homogeneous covering of BP membranes WO_3_ was further assessed by EDX spectroscopy. Figure 5 shows the EDX color mapping images of the border area between a WO_3_/BP membrane (lower area) and a BP membrane (i.e., the part of sputtered BP membrane, which was covered by a locking mask, upper area). BP membrane area looks like a red homogeneous region due to the presence of carbon and chemical impurities from SWNT, on the contrary WO_3_/BP membrane area looks like a red background covered by yellow and green spots, deriving by the covering of SWNT with WO_3_ nanoparticles.

**Figure 6:**
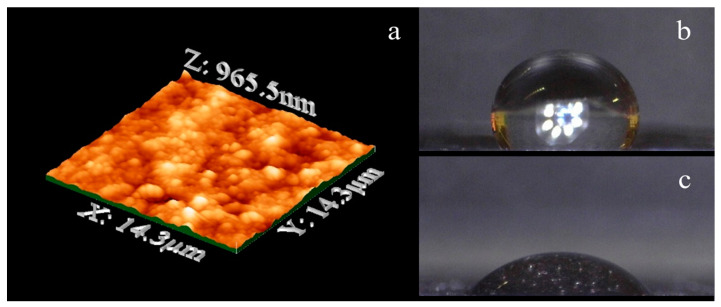
(**a**) AFM topology image of a WO_3_/BP membrane. The average rms roughness is 0.268 μm; (**b**) Average contact-angle value of a BP membrane; (**c**) Average contact-angle value of a WO_3_/BP membrane.

The WO_3_ layer showed an average rms roughness of 0.268 μm as determined by AFM measurements, Figure 6a. Such roughness gives a hydrophilic character to the top surface of WO_3_/BP membranes, as confirmed by contact-angle measurements. In fact, the average contact-angle value of WO_3_/BP membranes was found to be equal to 57.0° ± 0.5°, which is significantly smaller than the average contact-angle value (119.0° ± 0.5°) shown by a BP, Figure 6b,c. The contact angle values of the WO_3_/BP membranes did not change after 3 h continuous irradiation by solar simulator, confirming the stability of WO_3_ layer under UV-Vis light. The hydrophilicity of WO_3_/BP membranes could result in a possible increase of membrane fouling, i.e., the deposition of organic cakes onto the surfaces, but such possible drawback is overcome by fouling mitigation deriving from the photoactivity of WO_3_ layers. Moreover, the increase in hydrophilicity is expected to have a positive effect in the membrane permeation properties and makes such membranes suitable for the filtration of aqueous solutions.

It is well known that CVD deposition of tungsten trioxide onto substrates gives amorphous WO_3_, which is about ten times less photoactive than monoclinic WO_3_ [35]. Consequently, WO_3_/BP membranes were thermal treated at 350 °C in order to convert the amorphous WO_3_ layer into the more photoactive monoclinic one. Obviously, such treatment was possible thanks to the enhanced thermal properties of BP compared to polymer membranes.

**Figure 7:**
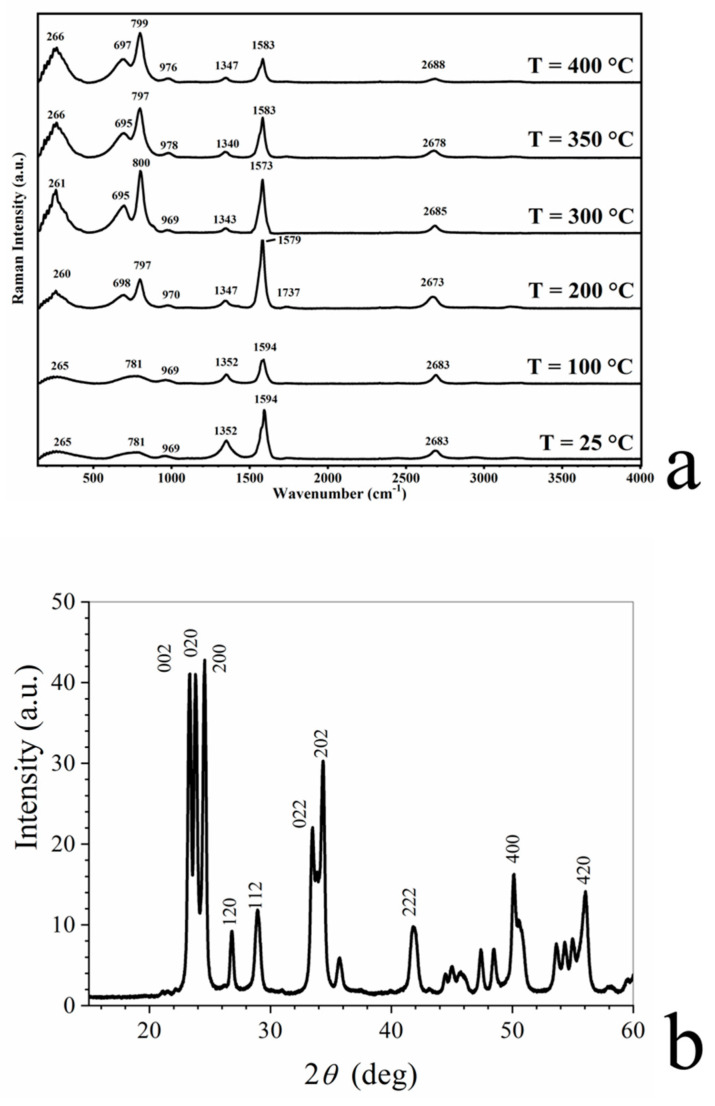
(**a**) Thermal evolution of Raman spectrum of WO_3_ deposited onto BP membranes. The peaks at around 700 and 800 cm^−1^ are assigned to WO stretching (ν), WO bending (δ), and OWO deformation (γ) modes, respectively, confirming the monoclinic structure of WO_3_; (**b**) X-ray diffraction patterns of WO_3_ layer sputtered onto BP membranes. Peaks are related to the reflection planes of the monoclinic phase of WO_3_.

Figure 7a shows the thermal evolution of micro-Raman spectrum of WO_3_/BP membranes. The Raman spectrum of as-deposited WO_3_ layer shows three main vibrational bands in the range of 200–1000 cm^−1^ observed at 265, 781, and 969 cm^−1^. The first peak increases in height as a function of the temperature, while the second splits into two intense peaks at around 700 and 800 cm^−1^. These peaks are the typical Raman peaks of monoclinic crystalline WO_3_, which correspond to the stretching vibrations of the bridging oxygen [36,37], and are assigned to WO stretching (ν), WO bending (δ), and OWO deformation (γ) modes, respectively [38,39]. 

The change of amorphous structure of WO_3_ to monoclinic WO_3_ was further confirmed by XRD as shown in Figure 7b, where the reported peaks are related to the reflection planes of the monoclinic phase of WO_3_.

The transmembrane flux WO_3_/BP membranes was evaluated in a small continuous plant and found to be 9.4 × 10^+3^ L m^−2^·h^−1^·bar^−1^, a value which is slightly lower (−25%) than pristine BP membrane as a consequence of the deposition of WO_3_ layer. 

The photoactivity of WO_3_/BP membranes was tested with model pollutant water solutions (250 mL) of a cationic dye (Methylene Blue, MB, 5, 10, and 20 ppm), an anionic dye (Indigo Carmine, IC, 20 ppm) and a drug (Diclofenac Sodium, DS, 20 ppm), which were recirculated by a peristaltic system through a round cell. 

**Figure 8:**
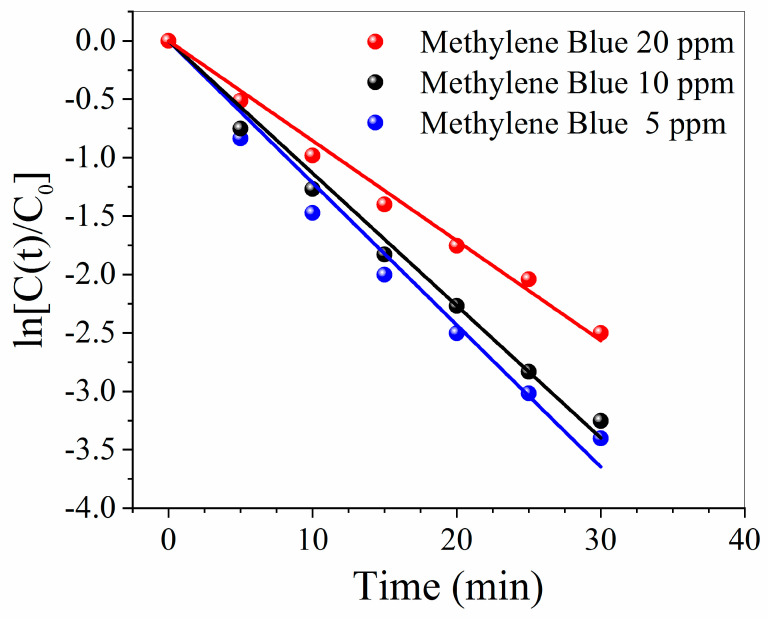
Effect of initial concentrations of Methylene Blue (5, 10, and 20 ppm) on the reaction rate.

The effect of initial concentrations of Methylene Blue (5, 10, and 20 ppm) on the reaction rate is shown in Figure 8. It is evident that the kinetic constant values decrease with increasing initial concentration (k values were 0.122 ± 0.003, 0.113 ± 0.003, and 0.085 ± 0.002 min^−1^, respectively). The higher values for the kinetic constant obtained at lower MB concentrations are explained as a consequence of [31,40]:

- The increase of the number of photons available per BM molecule;
- the higher amount of available catalytically active sites per BM molecule; and
- an easier penetration of photons through the less concentrated solutions.

Nevertheless, the reported rate constants were corrected for the different photon fluence and their values do not differ so much from the uncorrected ones (0.118 ± 0.003, 0.110 ± 0.002 and 0.082 ± 0.002 min^−1^, respectively, see SI). Accordingly, the different photon absorption from Methylene Blue solutions at different concentrations is not the major cause for the observed differences in the rate constants for Methylene Blue degradation. Most probably, such differences could arise from the competition of MB molecules towards active surface sites and reactive oxygen species [41]. 

In all cases the percentage of MB removal within 35 min was larger than 90%. The residual concentration of MB was respectively 0.3, 0.7, and 1.5 ppm, values in agreement with other data present in literature [40]. Removal experiments with no irradiation found very low pollutant adsorption by WO_3_/BP membranes. After a 3 h run a WO_3_/BP membrane was able to adsorb about 0.003 mg of MB, which was not a significant quantity compared to the weight amount of MB present in the used solutions. 

**Figure 9:**
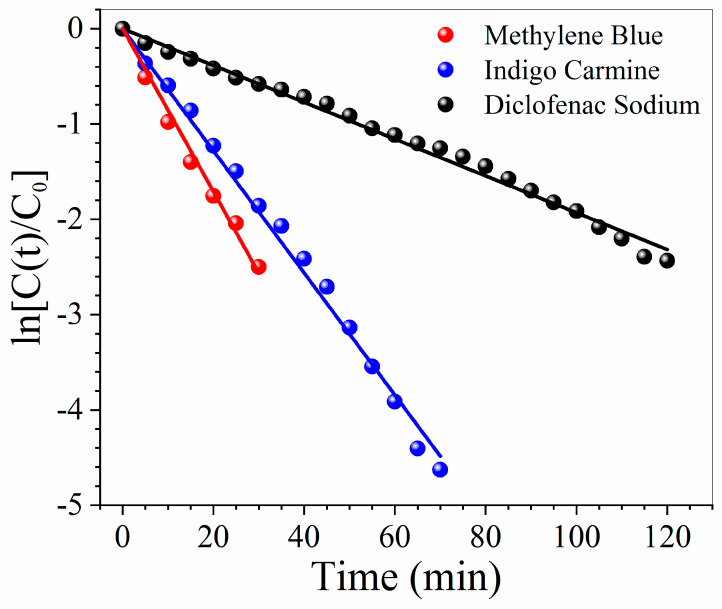
Photoactivity of WO_3_/BP membranes against water solutions of a cationic dye (Methylene Blue 20 ppm), an anionic dye (Indigo Carmine 20 ppm) and a drug (Diclofenac Sodium 20 ppm).

Figure 9 reports the photocatalytical properties of WO_3_/BP membranes against water solutions of a cationic dye (Methylene Blue 20 ppm), an anionic dye (Indigo Carmine 20 ppm) and a drug (Diclofenac Sodium 20 ppm), generally used as model pollutants. In all cases WO_3_/BP membranes are able to efficiently degrade the water contaminants with a kinetic constant value of 0.085 ± 0.002, 0.064 ± 0.001, and 0.019 ± 0.001 min^−1^, respectively. Such values are of the same order of magnitude or lower than the kinetic constants against the same pollutants, found with WO_3_, TiO_2_, or other catalyst nanoparticles dispersed either in the solutions or casted on carbon nanotubes, flakes of graphene oxide or porous polymer membranes [42,43,44,45,46,47,48,49,50], as no BP bearing photocatalysts, to the knowledge of authors, was ever proposed in literature.

Dark changes in absorbance were less than 1%, while UV controls for all three pollutant solutions found that after three hours of irradiation the absorbance changes due to the photolysis through a BP membrane were less than 2%. Such results can be explained by the particular spectrum of solar simulators (see Figure S1), which have only UV-A and UV-B emissions, and by the particular transmittance of N-BK7 optical glass cover, which cuts UV-B emissions with wavelengths lower than 300 nm. The absence of UV-C and lower UV-B wavelengths remarkably reduces the molecular degradation of pollutants by photolysis.

The performance of WO_3_/BP membranes was also compared with the photoactivity of 0.2 mg of monoclinic WO_3_ nano-powder (the same amount of WO_3_ sputtered onto BP membranes).

**Figure 10:**
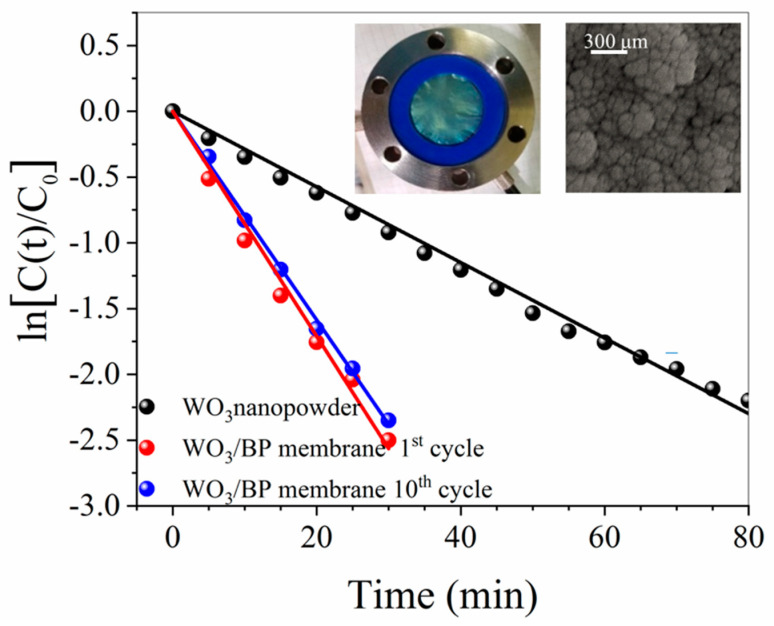
Photodegradation of Methylene Blue by a WO_3_/BP membrane (red dots) and by monoclinic WO_3_ nanopowder (black dots) in the same amount of WO_3_ present on WO_3_/BP membrane.

As shown in Figure 10, the photodegradation of Methylene Blue by both systems (WO_3_/BP membrane and monoclinic WO_3_ nano-powder) follows a first order kinetics with a rate constant of 0.085 ± 0.002 min^−1^ and 0.029 ± 0.001 min^−1^, respectively. The enhancement of the photoactivity in WO_3_/BP membranes can be due to the presence of BP, as the SWNT substrate prevents the electron/hole pair recombination during photocatalysis and increases the kinetic rate constant [51]. An almost complete photodegradation of Methylene Blue was obtained within ≈ 50 min and ≈ 140 min by using WO_3_/BP membranes and WO_3_ nano-powder, respectively. Such relatively short degradation times make the WO_3_/BP membranes suitable for applications in advanced oxidation processes. In addition, the long-term stability of WO_3_/BP membranes was checked by ten cycles of successive photocatalysis processes. Figure 10 shows the morphology and the photocatalycal efficiency of a WO_3_/BP membrane after the tenth photocatalytical cycle of a 20 ppm BM solution, revealing no evident damage in the morphology of WO_3_/BP membranes and no important change in the degradation efficiency, being the rate constant value equal to the pristine one within experimental errors (0.081 ± 0.002 min^−1^). In addition, the morphology of the WO_3_/BP membrane, after the tenth photocatalytical cycle, reveals the absence of any cakes on the surface and a morphology similar to that shown in the pristine WO_3_/BP membrane (Figure 3b), thanks to its photoactivity and different surface chemistry preventing and destroying any deposition. Further investigations are in progress to test the antifouling activity of WO_3_/BP membranes on real industrial wastes rather than on model dye and drug water solutions. 

## 4. Conclusions

A new flexible membrane, based on SWNT and with improved thermal and catalytic properties, was obtained by RF magnetron sputtering of a nanostructured thin layer of tungsten trioxide and successive conversion in the more photoactive monoclinic phase. The WO_3_/BP membrane was characterized by SEM, TGA, porosimetry, XRD, EDX, AFM, Raman, contact angle and permeation measurements. The photocatalytic activity of WO_3_/BP membranes was tested following the degradation of three different pollutant water solutions in a small continuous plant confirming the beneficial contribution of the hydrophilic WO_3_ layer. The degradation kinetics rate of the Methylene Blue by WO_3_/BP membranes was about three times that found by an equivalent amount of WO_3_ nano-powder. 

The main advantages of the proposed WO_3_/BP membranes can be summarized in:

1. The possibility to make heterogeneous photocatalytical processes with an easier catalyst recovery and reuse;
2. their application in continuous flow plants;
3. a simpler and cleaner synthetic approach. Chemical vapor deposition processes do not require long and expensive purification procedures, which are necessary in other chemical syntheses, such as solvo-thermal processes. In addition, CVD allows the catalyst amount saving, avoiding its dispersion in the substrate bulk;
4. a higher photocatalytical efficiency, due to the facilitated electron-transfer between carbon nanostrucutres and catalyst nanoparticles, a reduced recombination between electrons and holes [51], and the presence of catalyst nanoparticles with small size just only on the top surface of substrates rather than in the polymer bulk (where they cannot play any catalytic action);
5. the possibility to change the photocatalyst crystal structure in a more photoactive one by thermal annealing processes at temperatures higher than the melting point of commonly used polymer substrate. PTFE, polytetrafluoroethylene, which has one of the highest melting points, melts at 327 °C, a temperature lower than the WO_3_ amorphous-monoclinic phase transition temperature. On the contrary, BP membranes result thermally stable up to 400 °C;
6. BPs have both light weight and strong mechanical resistance, and, consequently, are easy to handle. In addition, BPs are resistant to all organic solvents and acid and base solutions, while porous polymer membranes can be damaged; and
7. a green chemisty approach with an almost zero environmental footprint, as the BP preparation is based on rather simple and clean experimental set-ups, which allow the recovery and reuse of solvents, CNT processing waste, end of life BPs and photocatalysts for the preparation of new catalyst/BP membranes.

The improved photoactivity, long-term stability, solvent-free features, fast catalyst recovery and re-use, and the possibility of an easy up-scale make WO_3_/BP membranes efficient devices for the pollutant degradation by advanced oxidation processes.

## Figures and Tables

**Table 1:** Physical-chemical properties of BPs.

Property	Value
Thickness	45 ± 2 μm
Diameter	37.0 ± 0.1 mm
Density	0.60 ± 0.03 g·cm^−3^
Porosity	70 ± 5 %
Electrical Conductivity	83 ± 4 S cm^−1^
Tensile strength	11.8 ± 2.2 MPa
Fracture Strain	2.6 ± 0.1%
Young’s Modulus	0.9 ± 0.1 GPa
Water Flow Rate	12,500 ± 100 L m^−2^·h^−1^·bar^−1^

## Supplementary Materials

The following are available online at https://www.mdpi.com/2077-0375/10/7/157/s1, Figure S1: Spectrum of Suntest CPS+ sun simulator for three different irradiance values. Reprinted from [S1], with permission from Royal Society of Chemistry, Figure S2: Normalized fluence across solutions of Methylene Blue, Indigo Carmine, and Diclofenac Sodium, Table S1: Fluence uncorrected and fluence corrected rate constants.
