# Supplementary material for: WO3/Buckypaper Membranes for Advanced Oxidation Processes
Source: Membranes (Basel). 2020 Jul 20;10(7):157. doi: 10.3390/membranes10070157 (PMC7407767; doi:10.3390/membranes10070157)
Supplement: Supplementary file 1 [file membranes-10-00157-s001.pdf]

membranes

Supporting Information

WO3/Buckypaper Membranes for Advanced Oxidation Processes

Giovanni De Filpo [1,*], Elvira Pantuso [2], Aleksander I. Mashin [3], Mariafrancesca Baratta [1] and Fiore Pasquale Nicoletta [2,*]

[1] Department of Chemistry and Chemical Technologies, University of Calabria, 87036 Rende (CS), Italy; mariafrancesca.baratta@unical.it
[2] Department of Pharmacy, Health and Nutritional Sciences, University of Calabria, 87036 Rende (CS), Italy; elvirapnt.ep@gmail.com
[3] Applied Physics & Microelectronics, Lobachevsky State University of Nizhni Novgorod, 603950 Nizhni Novgorod, Russian Federation; mashin@unn.ru
* Correspondence: giovanni.defilpo@unical.it (G.D.F.); fiore.nicoletta@unical.it (F.P.N.); Tel.: +39-0984-492095 (G.D.F.), +39-0984-493194 (F.P.N.)

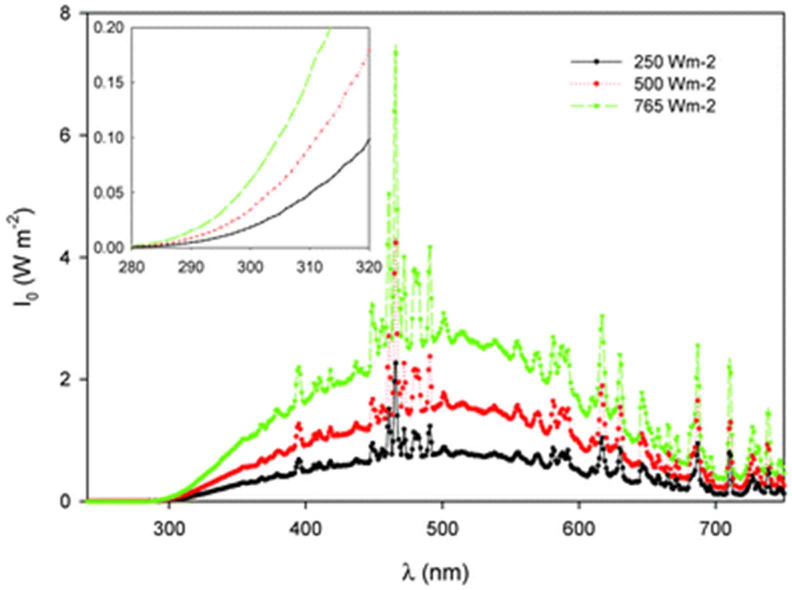

Figure 1. Spectrum of Suntest CPS+ sun simulator for three different irradiance values. Reprinted from [1], with permission from Royal Society of Chemistry.

Fluence Normalization of Kinetic Observations

The activation wavelength of WO_3 (2.6 eV → 476.86 nm ≈ 477 nm) was selected on a FieldMaxII-TO digital power/energy meter (Coherent Italia S.r.l., Monza, Italy) equipped with a PM10 thermopile. Then, the fluence after crossing a 5 mm thick cell containing water (reference) and solutions of Methylene Blue, Indigo Carmine and Diclofenac Sodium at different concentrations (2.5 ppm, 5 ppm, 10 ppm, 15 ppm, and 20 ppm) was measured. The active surface area was fixed in 1 cm^2 by an aluminium mask.

Results are reported in the following Figure S2, where the fluence across dye solutions was normalized to the fluence across water.

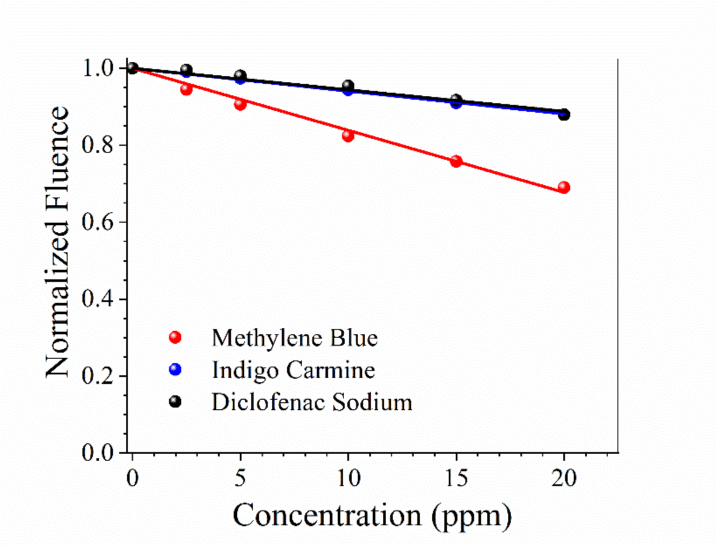

Figure S2. Normalized fluence across solutions of Methylene Blue, Indigo Carmine, and Diclofenac Sodium.

The normalized fluences, NF, were well fitted as function of concentration (ppm) with linear fits ($R^2 > 0.999$) with fixed intercept:
Methylene Blue: NF = 1 - 0.0161 ppm
Indigo Carmine: NF = 1 - 0.0059 ppm
Diclofenac Sodium: NF = 1 - 0.0056 ppm
and used to normalize the kinetic observations.

The rate constants are reported in the following Table S1.

Table S1. Fluence uncorrected and fluence corrected rate constants.

Solution	Uncorrected Rate Constant (min^{-1})	Corrected Rate Constant (min^{-1})
Methylene Blue (5 ppm)	0.118 ± 0.003	0.122 ± 0.003
Methylene Blue (10 ppm)	0.110 ± 0.002	0.113 ± 0.003
Methylene Blue (20 ppm)	0.082 ± 0.002	0.085 ± 0.002
Indigo Carmine (20 ppm)	0.062 ± 0.001	0.064 ± 0.001
Diclofenac Sodium (20 ppm)	0.018 ± 0.001	0.019 ± 0.001
WO_3 nanopowder	0.028 ± 0.001	0.029 ± 0.001
Methylene Blue (20 ppm) 10th run	0.080 ± 0.002	0.081 ± 0.002

The corrected values of rate constants do not differ so much from the uncorrected ones and exclude that the different photon absorption from Methylene Blue solution at different concentrations could be the major cause for the observed differences in the rate constants for Methylene Blue degradation. Most probably, such differences could arise from the competition of MB molecules towards active surface sites and reactive oxygen species [2].

References

1. Weber, J.; Halsall, C.J.; Wargent, J.J.; Nigel D. Paul, N.D. A comparative study on the aqueous photodegradation of two organophosphorus pesticides under simulated and natural sunlight. *J. Environ. Monit.* **2009**, *11*, 654–659. DOI: 10.1039/b811387d

2. Wang, W.-Y., Ku, Y. Photocatalytic degradation of Reactive Red 22 in aqueous solution by UV-LED radiation. *Water Res.* **2006**, *40*, 2249–2258. DOI: 10.1016/j.watres.2006.04.041
